# Commentary: Detection Methods for Autoantibodies in Suspected Autoimmune Encephalitis

**DOI:** 10.3389/fneur.2019.00202

**Published:** 2019-03-11

**Authors:** Avi Gadoth

**Affiliations:** Autoimmune Encephalitis and Paraneoplastic Syndromes Clinic, Department of Neurology, Tel-Aviv Medical Center, Tel-Aviv, Israel

**Keywords:** PCA-2, MAP1B, paraneoplastic, autoimmune, small cell lung cancer

In their paper, Recken and colleagues, review the different autoantibodies, related clinical presentations and cancer associations and detection methods for autoantibodies. The review was comprehensive and nicely described most of the established antibodies. However, when discussing paraneoplastic associated antibodies, the authors failed to mention Purkinje cell antibody type 2 (PCA-2) which is a well-established paraneoplastic antibody (1). PCA-2 has first been described in 2000 by Vernino and Lennon (2) that reported 10 patients with diverse clinical presentations of which 80% had lung cancer. Recently it was discovered that this antibody targets the microtubule associated protein 1B (MAP1B) (3). MAP1B is a part of the microtubule associated protein family that also includes MAP1 (A and B), MAP2 (A and B), and tau protein. These proteins bind and stabilize microtubules. MAP1B expression peaks during early stages of neuronal development and plays an important role in neuronal differentiation, including dendritic spine formation and synaptic maturation (4).

In this recent paper, describing 118 patients, PCA-2 was shown to be as common as anti amphiphysin IgG and more common than ANNA-2 (also known as anti-Ri) and PCA-Tr (also known as delta/notch-like epidermal growth factor-related receptor [DNER]). PCA-2 positivity was associated with cancer in 79% of the patients, with the majority being small cell lung cancer (SCLC) (3).

The clinical presentation among patients varied and included peripheral neuropathy, 53%; cerebellar ataxia, dysmetria, or dysarthria, 38%; and encephalopathy, 27%. Ophthalmologic and spinal involvements were also reported.

MAP1B was shown to present in SCLC as well as in the brain which further support it as a paraneoplastic marker.

The fact that PCA-2 is not yet available in commercial kits, might mistakenly rule out the diagnosis of a paraneoplastic neurological syndrome. This makes including PCA-2 in such a review even more important so it will remain in the differential diagnosis in cases the available commercial kits are negative. In such cases it is important to seek for cancer and if possible use immuno-fluorescence assays available for PCA-2 detection.

## Author Contributions

The author confirms being the sole contributor of this work and has approved it for publication.

### Conflict of Interest Statement

The author published a paper in which MAP1B was described as the antigen of PCA-2. The author is a part of the group that holds a patent on MAP1B as a marker for SCLC and paraneoplastic neurological autoimmunity – patent number 2016-372.
